# Radiation therapy enhanced therapeutic efficacy of anti-PD1 against gastric cancer

**DOI:** 10.1093/jrr/rraa077

**Published:** 2020-09-22

**Authors:** Sen Hong, MiaoMiao Bi, HaiYao Yu, ZhenKun Yan, HeLei Wang

**Affiliations:** Department of Colorectal and Anal Surgery, The First Hospital of Jilin University, Changchun 130021, P.R. China; Department of Ophthalmology, China-Japan Union Hospital of Jilin University, Changchun 130033, P.R. China; Department of Chief Pharmacist, Changchun Food and Drug Inspection Center, Changchun 130033, P.R. China; Endoscopy Center, China-Japan Union Hospital of Jilin University, Changchun 130033, P.R. China; Department of Gastrointestinal Surgery, The First Hospital of Jilin University, Changchun 130021, P.R. China

**Keywords:** radiation therapy, gastric cancer, anti-PD1, immunotherapy; intratumor T cells

## Abstract

Radiation therapy is an important method in tumor treatment with distinct responses. This study aimed to investigate the immune effects of radiation therapy on the syngeneic gastric tumor model. Mouse forestomach carcinoma (MFC) cells were irradiated with different X-ray doses. Cell proliferation was determined by clonogenic assay. Gene and protein expression were determined by real-time quantitative PCR and western blot, respectively. The tumor model was established by subcutaneously injecting tumor cells in 615-(H-2 K) mice. Levels of immune-related factors in tumor tissues were determined by immunohistochemistry and flow cytometry. 5 Gy × 3 (three subfractions with 4 h interval) treatment significantly inhibited cell proliferation. Protein expression of stimulator of interferon genes (Sting) and gene expression of *IFNB1, TNFα* as well as *CXCL-9* significantly increased in MFC cells after irradiation. In the MFC mouse model, no obvious tumor regression was observed after irradiation treatment. Further studies showed Sting protein expression, infiltration of dendritic cells and T cells, and significantly increased PD-1/PD-L1 expression in tumor tissues. Moreover, the irradiation treatment activated T cells and enhanced the therapeutic effects of anti-PD1 antibody against MFC tumor. Our data demonstrated that although the MFC tumor was not sensitive to radiation therapy, the tumor microenvironment could be primed after irradiation. Radiation therapy combined with immunotherapy can greatly improve anti-tumor activities in radiation therapy-insensitive tumor models.

## INTRODUCTION

As the fourth (in males) and fifth (in females) most common cause of cancer-related deaths in the world, the majority of patients with gastric cancer are often diagnosed in the advanced stage, which is not curable with a median survival time of 8–10 months. Therefore, it is important to explore the use of multiple modalities in order to improve the therapeutic ratio and overcome inherent resistance [1, 2].

As an important method in tumor therapy, radiation causes DNA damage by directly or indirectly ionizing atoms in DNA double strands in tumor cells [3]. More than 70% of patients with cancer need radiation therapy during clinical treatment, but some tumor types are less sensitive, requiring the development of novel combinatorial treatments [4]. DNA damage caused by radiation therapy in mammalian cells activates cytoplasmic DNA receptors and further activates the stimulator of interferon genes (Sting) signaling pathway, which is essential for radiation-induced anti-tumor responses. As a stimulatory factor, Sting also activates type I interferon (IFN) gene transcription via the STING/TANK-binding kinase (TBK)/interferon regulatory factor 3 (IRF3)/Nuclear Factor kappa-light-chain-enhancer of activated B cells (NF-kB) signaling pathway [5, 6]. IFN produced by dendritic cells (DCs) helps to establish a critical link between innate and adaptive immunity, through upregulating major histocompatibility complex (MHC) class I molecules on cells, promoting maturation and activation of DCs, and eventually leading to Th1 activation [7, 8]. High levels of type I IFN can directly affect the activation and differentiation of T cells, but it can also regulate the response of T cells by affecting DCs [9]. Studies showed that radiation therapy can promote the effective activation of DCs in several ways, including increasing their infiltration in tumor cells and enhancing the production of type I IFN in tumor tissues [10]. Irradiation treatment can also upregulate the expression of CD70 on DCs, enhance the activation of T cells, induce the expression of chemokines CXCL-9, CXCL-10, CXCL-11 as well as CXCL-16, and attract antigen-specific T cells to infiltrate irradiated tumor regions [11, 12].

Programmed death-ligand 1 (PD-L1) is a key mechanism of immunosuppression in a variety of tumors and an important predictor of programmed cell death protein 1 (PD-1)/PD-L1 blockade reactivity. IFN can increase the expression of PD-L1 on various tumor cells [13, 14]. Inhibition of the PD-1/PD-L1 pathway has been shown to be associated with strong anti-tumor activity in mouse tumor models and clinical trials [15, 16].

In summary, radiation therapy combined with immunotherapy may be an effective strategy for the treatment of cancers. However, the molecular mechanism by which radiation therapy activates innate immunity and then induces T cell immune responses is not well understood. This study evaluated the immune responses in the tumor microenvironment induced by X-rays in a syngeneic gastric tumor model, providing a theoretical basis for the combined treatment regimen.

## MATERIALS AND METHODS

### Experimental animals

Six-week old female 615-(H-2 K) mice were purchased from the Chinese Academy of Sciences (Beijing, China) and maintained under specific pathogen-free conditions. Mice had free access to food and water during the experimental period. All experiments were approved by the Institutional Animal Care and Use Committees of Jilin University.

### Cell culture

The mouse forestomach carcinoma (MFC) cell line was purchased from the Chinese Academy of Sciences (Beijing, China). Cells were cultured in RPMI 1640 complete medium supplemented with 10% fetal bovine serum (FBS), 100 U/L penicillin and 100 mg/L streptomycin at 37°C, 5% CO_2_. 1 × 10^6^ cells were seeded in the culture dish and irradiated (X-RAD225, SSD 50 cm, filter 2 mm) with doses of 0, 5, 10, 20 Gy and 5 Gy × 3 (three subfractions with 4 h interval). After irradiation, cells were cultured for 48 h and then collected for further analysis.

### Clonogenic assay

Cells in the log phase were plated into 25-ml cell culture flasks and irradiated with doses of 0, 5, 10, 20 Gy and 5 Gy × 3. Cells were then counted and five different numbers of cells per group were seeded in triplicates into tissue culture dishes (Greiner Bio-One, Frickenhausen, Germany) containing fresh culture medium. Colonies were allowed to form over 2–3 weeks. The cell culture medium was removed and cells were washed twice with phosphate-buffered saline (PBS). Colonies were fixed in 100% methanol for 30 min and stained with Giemsa for 15 min. The number of colonies containing >50 cells was determined and the surviving fraction was calculated.

### Western blot

The total protein was extracted with lysis buffer and the protein concentration was determined by the bicinchoninic acid assay (BCA) method. Samples with 40 μg of protein were used for 4–12% sodium dodecyl sulfate-polyacrylamide gel electrophoresis and protein was then transferred to a polyvinylidene difluoride membrane. After blocking with 5% skim milk powder for 1 h, the membrane was incubated with primary antibodies (anti-Sting, rabbit pAb, ab179775, 1:500; anti-β-actin, rabbit pAb, ab1801, 1:3000) at 4°C overnight. After washing, the membrane was incubated with horseradish peroxidase-labeled antibody (goat anti-rabbit IgG H&L HRP, ab6721, 1:3000) for 1 h at room temperature. The membrane was washed three times and visualized by the enhanced chemiluminescence system.

### Real time-PCR

Total RNA was extracted with 1000 μL of TRIzol reagent. Total RNA (0.5 μg) was used to generate complementary DNA with SuperScript master mix (Bio-Rad, CA). Quantitative PCR (qPCR) was performed on a StepOne system using SYBR green supermix (ThermoFisher, MA) with the comparative Ct value method to quantify the expression of genes of interest in different samples. The mRNA levels were normalized to the housekeeping gene *β-actin*. The gene-specific primer sequences were as follows: *IFNB1*, forward: ccctatggagatgacggaga; reverse: ctgtctgctggtggagttca, NCBI reference: NM_010510.1; *TNFα*, forward: agcccccagtctgtatcctt; reverse: ctccctttgcagaactcagg, GenBank: M38296.1; *CXCL-9*, forward: aatttcatcacgcccttgag; reverse: tctccagcttggtgaggtct, NCBI reference: NM_008599.4; *IL-10*, forward: ccagggagatcctttgatga; reverse: cattcccagaggaattgcat, NCBI reference: NM_010548.2; *Mage-A1*, forward: atggctgactcccgtaacac; reverse: tcctcagatgggctatcagg, NCBI reference: NM_020015.2; *Mage-A3*, forward: atgaccaggagaccatggag; reverse: aggccctctgatcctttgat, NCBI reference: NM_020017.2; *β-actin*, forward: tgttaccaactgggacgaca; reverse: ggggtgttgaaggtctcaaa, NCBI reference: NM_007393.5.

### Mouse tumor model and treatment

MFC cells (5 × 10^5^) were subcutaneously inoculated into 615 mice. When the tumor volume reached 50–100 mm^3^, tumor-bearing mice were divided into two groups (five mice per group) based on the tumor volume: control group (0 Gy) and irradiation group (5 Gy × 3) and treatment was started. For irradiation, mice were immobilized via a single injection of ketamine (80 mg/kg) and xylazine (16 mg/kg). Irradiation was delivered to the mouse tumor using an irradiator (RT 250; Philips Medical Systems, Eindhoven, The Netherlands) at 250 kV and 12 A with filtration of 0.4 mm of tin and 0.25 mm of copper. The radiation dose was delivered at a rate of 1 ± 0.1 Gy per min. Lead shielding was used to limit radiation exposure to other areas of the body. Three 5 Gy doses with a 4 h interval were administered for a total radiation dose of 15 Gy. The diameter in two dimensions (length and width) of tumors was measured twice weekly using a caliper to calculate the tumor volume (*V*) with the formula *V* = length x width^2^/2. All mice were sacrificed to collect tumor samples for analysis when the tumor volume of any mouse reached 1500 mm^3^.

In the combination treatment experiments, 5 × 10^5^ MFC cells were inoculated into 615 mice subcutaneously. When the tumor volume reached 50–100 mm^3^, mice were divided into four groups (five mice per group) and tumor-bearing mice were treated with IgG (BP0089, BioXcell), anti-PD1 antibody (5 mg/kg, once a week, I.P., BP0273, BioXcell), irradiation (5 Gy x 3), or the combination treatment with irradiation (5 Gy x 3) first and then anti-PD1 antibody (5 mg/kg, once a week, intraperitoneal (IP)). The irradiation was the same as described above. All mice were sacrificed to collect tumor samples for analysis when the tumor volume of any mouse reached 1500 mm^3^.

### Immunofluorescence

Tumor tissues were fixed in 4% paraformaldehyde for 4 h at 4°C and then processed through a graded series of sucrose in PBS (10% for 1 h, 20% for 1 h and 30% overnight) at 4°C. After being embedded in optimum temperature cutting compound, samples were frozen on dry ice and stored at −80°C. Thick frozen sections (10 μm) were cut sagittally and placed onto slides. Sections were incubated in PBS containing 5% normal goat serum, 1% bovine serum albumin (BSA) and 0.5% Triton X-100 for 1 h, followed by incubation with primary antibodies (anti-CD3 FITC, Clone 17A2 and anti-CD8 PE, Clone 53–6.7, BioLegend, San Diego, CA, USA) overnight at 4°C. After washing with PBS, sections were mounted with VECTASHIELD mounting medium with 4′,6-diamidino-2-phenylindole (DAPI) and visualized under a fluorescence microscope (Olympus, Shinjuku, Japan).

### Flow cytometry

Tumor tissue was harvested and cut into small fragments followed by digestion with a tumor disassociation kit (Miltenyi Biotec, USA) for 30 min, and then filtered by 70 μm cell strainers. Cells were stimulated with phorbol myristate acetate (PMA)/ionomyocin in the presence of Golgi stop and Monesin (eBioscience) for 4 h. Cells were washed with PBS and then stained with Live/Dead dye and fluorochrome-labeled antibodies specific to cell surface markers [anti-CD45 Alexa 700 (Clone 30-F11, BioLegend), anti-PD-L1 PE-Cy7 (Clone 10F.9G2, BioLegend), anti-CD3 PerCP (Clone 45-2C11, BioLegend), anti-CD8 APC-Cy7 (Clone 53–6.7, BD), anti-CD11c FITC (ab210308, Abcam), anti-PD1 PE (Clone RMP1–30, BioLegend), anti-CD4 PE-CF594 (Clone RM4–5, BD), anti-MHC-I PE (Clone 28–14-8, BioLegend), anti-CD44 APC-Cy7 (Clone IM7, BioLegend), anti-CD62L APC (Clone MEL-14, BioLegend), anti-CD25 PerCP (Clone 3C7, BioLegend), anti-CD127 FITC (Clone A7R34, BioLegend), anti-ICOS PE-Cy7 (Clone 7E.17G9, BioLegend)]. After fixation–permeabilization, cells were stained with fluoro-conjugated antibodies specific to intracellular markers [anti-Ki67 APC (Clone 16A8, BioLegend), anti-IFNγ APC (Clone XMG1.2, BioLegend), anti-granzyme B FITC (Clone GB11, BioLegend)] or with the isotype control. Flow cytometry analysis was performed on BD LSRFortessa and data were analyzed by FlowJo V10.

### Statistical analysis

Statistical analysis was carried out using the Prism software (GraphPad). Measurement data between two groups were compared using Student’s t-test; measurement data among multiple groups were compared using one-way analysis of variance followed by Dunnett’s t-test. *P* < 0.05 was considered as significant difference.

## RESULTS

### Irradiation treatment inhibited MFC cell proliferation and up-regulated expression of immune-related factors

**Fig. 1.**
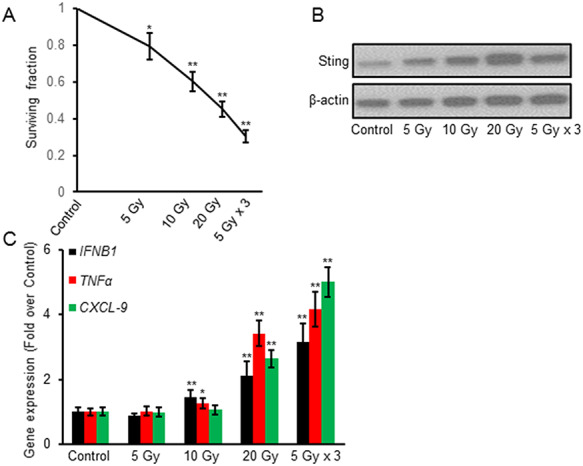
Irradiation inhibited MFC cell proliferation and increased immune-related factor expression. MFC cells were irradiated and cell proliferation was examined by clonogenic assay. The irradiation treatment decreased the surviving fraction (**A**). Irradiation activated an immunological reaction in MFC cells by protein expression of Sting (**B**) and mRNA levels of *IFNB1, TNFα, CXCL-9* (**C**). Data are expressed as mean ± SD (*n* = 3). ^*^P < 0.05; ^**^P < 0.01 vs Control.

MFC cells were irradiated with 0, 5, 10, 20 Gy and 5 Gy × 3. Results showed that irradiation treatment could significantly inhibit cell proliferation, shown by the clonogenic assay (Fig. 1A). Western blot showed the expression of Sting protein up-regulated after irradiation (Fig. 1B). The irradiation treatment also significantly increased gene expression of *IFNB1*, *TNFα* and the Sting activation marker *CXCL-9* (Fig. 1C).

**Fig. 2.**
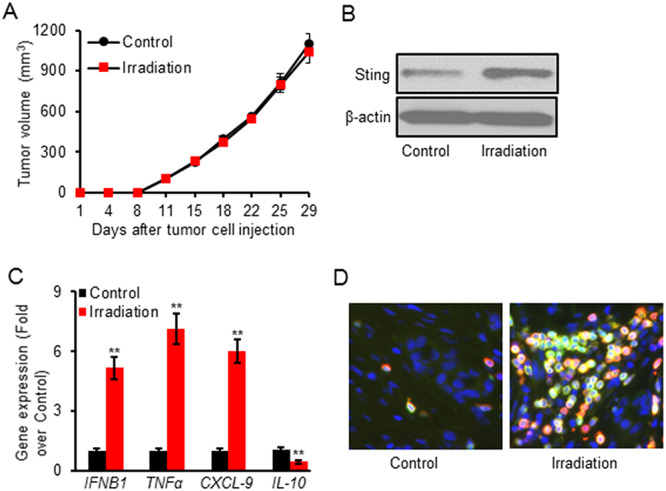
Irradiation alone did not delay tumor growth. MFC tumor-bearing mice were treated with Control (0 Gy) or Irradiation (5 Gy x 3). Tumor volume was measured twice weekly. All mice were sacrificed to collect tumor samples for analysis when the tumor volume of any mouse reached 1500 mm^3^. Irradiation did not obviously delay tumor growth (**A**). However, irradiation treatment primed the tumor microenvironment by increasing protein expression of Sting (**B**), up-regulating mRNA levels of *IFNB1, TNFα* as well as *CXCL-9* and down-regulating mRNA level of *IL-10* (**C**) and enhancing infiltration of CD8^+^ cells (**D**) in tumor tissues. Data are expressed as mean ± SD (*n* = 5). ^**^P < 0.01 vs Control.

### Irradiation treatment alone did not delay tumor growth

Irradiation treatment was confirmed to inhibit cell proliferation *in vitro*. Its anti-tumor effects were further evaluated in the mouse tumor model. A syngeneic gastric tumor model was established by injecting 5 x 10^5^ MFC cells subcutaneously into 615 mice. MFC tumor-bearing mice were treated with Control (0 Gy) or Irradiation (5 Gy x 3). Mice were sacrificed to collect tumor samples for analyses when the tumor volume reached 1500 mm^3^. No obvious tumor regression was observed after irradiation in the MFC mouse model (Fig. 2A), suggesting MFC tumor was not sensitive to radiation therapy. However, irradiation treatment increased Sting protein expression (Fig. 2B). qPCR results showed irradiation treatment significantly increased the gene expression of *IFNB1*, *TNFα* as well as *CXCL-9* and decreased immunosuppression gene interleukin 10 (IL-10) (Fig. 2C). The infiltration of CD8^+^ T cells also increased in tumor tissues after irradiation (Fig. 2D).

**Fig. 3.**
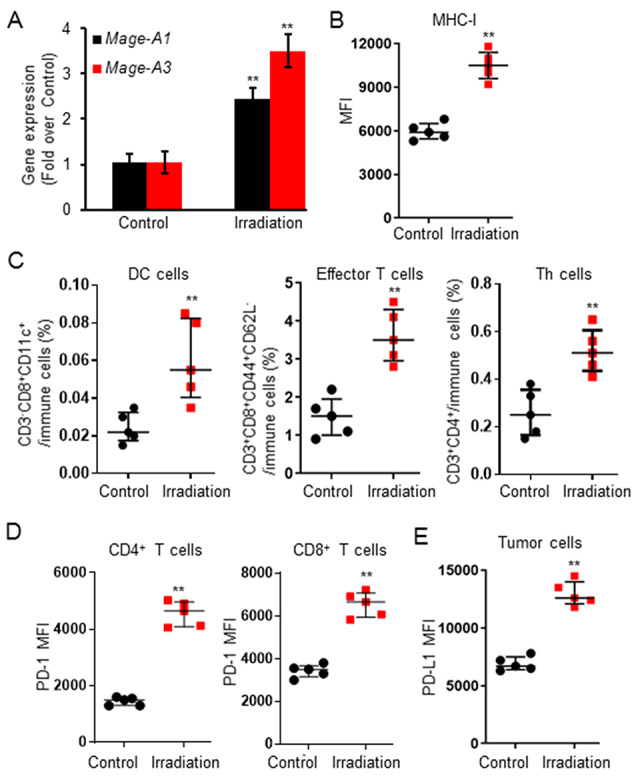
Irradiation treatment primed the tumor microenvironment in an MFC tumor model. MFC tumor-bearing mice were treated with Control (0 Gy) or Irradiation (5 Gy x 3). All mice were sacrificed to collect tumor samples for analysis when the tumor volume of any mouse reached 1500 mm^3^. Irradiation treatment enhanced antigen presentation by increasing gene expression of *Mage-A1* as well as *Mage-A3* (**A**) and MHC-I expression (**B**). Irradiation treatment promoted tumor infiltration of DC, effector T and Th cells (**C**). Irradiation treatment also increased PD-1 expression on intratumor CD4^+^/CD8^+^ T cells (**D**) and PD-L1 expression on tumor cells (**E**) in the MFC tumor tissues. Data are expressed as mean ± SD or median ± interquartile (*n* = 5). ^**^P < 0.01 vs Control.

### Irradiation treatment primed tumor microenvironment

**Fig. 4.**
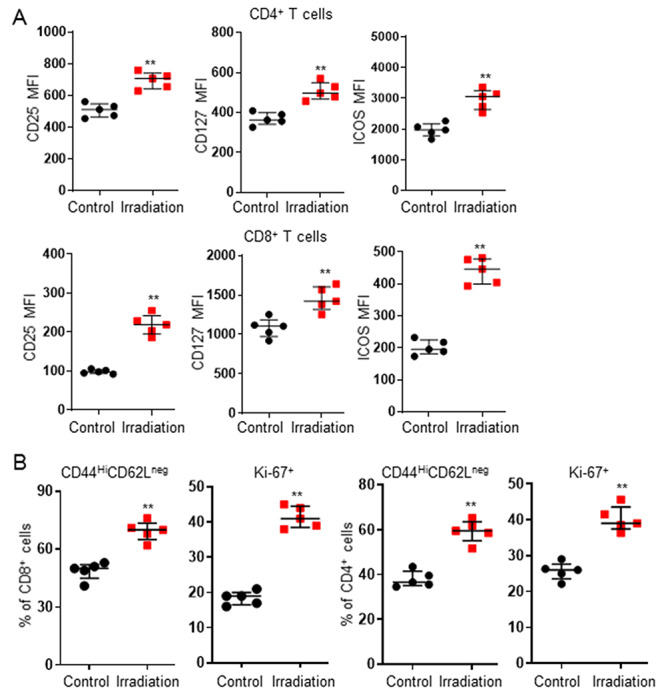
Irradiation treatment stimulated T cell function, activation and proliferation. MFC tumor-bearing mice were treated with Control (0 Gy) or Irradiation (5 Gy x 3). All mice were sacrificed to collect tumor samples for analysis when the tumor volume of any mouse reached 1500 mm^3^. Up-regulation of CD25, CD127 and ICOS was found in intratumor CD4^+^/CD8^+^ T cells after irradiation treatment (**A**). Irradiation treatment improved T cell activation (CD44^Hi^CD62L^neg^) and proliferation (Ki-67^+^) (**B**). Data are expressed as median ± interquartile (*n* = 5). ^**^P < 0.01 vs Control.

Further analysis of the tumor tissues showed irradiation treatment significantly increased the gene expression of neoantigen *Mage-A1* as well as *Mage-A3* (Fig. 3A) and the expression of MHC class I (Fig. 3B). Moreover, flow cytometry analysis (see online supplementary material for a gating strategy figure) showed a significant increase in the tumor infiltration of DC cells (CD3^+^CD8^+^CD11c^+^), Teff cells (CD3^+^CD8^+^CD44^+^CD62L^−^) and Th cells (CD3^+^CD4^+^) after irradiation treatment (Fig. 3C). Irradiation also significantly increased expression of PD-1 on intratumor CD4^+^/CD8^+^ T cells and PD-L1 on tumor cells in tumor tissues (Fig. 3D and E). Furthermore, an upregulation of CD25, CD127, ICOS was found in intratumor CD4^+^/CD8^+^ T cells after irradiation treatment (Fig. 4A). Irradiation treatment promoted CD8^+^/CD4^+^ T cell activation (CD44^Hi^CD62L^neg^) and proliferation (Ki-67^+^) (Fig. 4B). Our results indicated that irradiation treatment significantly increased T cell capacities of activation, proliferation and cytokine-production.

### Irradiation treatment enhanced therapeutic effects of anti-PD1

**Fig. 5.**
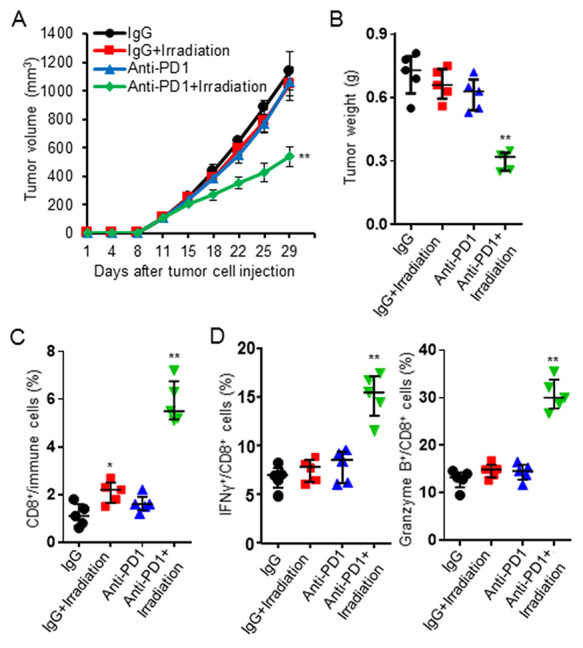
Irradiation synergized with anti-PD1 treatment to delay tumor growth. MFC tumor-bearing mice were treated with IgG, anti-PD1 antibody (5 mg/kg, once a week, I.P.), irradiation (5 Gy x 3), or the combination treatment. Tumor volume was measured twice weekly. All mice were sacrificed to collect tumor samples for analysis when the tumor volume of any mouse reached 1500 mm^3^. The combination treatment promoted tumor growth control (**A**) and decreased the tumor burden (**B**). Combination treatment significantly enhanced anti-tumor immunity by increasing CD8^+^ infiltration in tumor tissues (**C**) and producing more IFNγ as well as granzyme B in tumor tissues (**D**). Data are expressed as mean ± SD or median ± interquartile (*n* = 5). ^*^P < 0.05, ^**^P < 0.01 vs IgG group.

Irradiation treatment improved T cell infiltration and function, but also up-regulated PD-1/PD-L1 expression, suggesting that irradiation might synergize with anti-PD1 blockade to induce long-term tumor control. Tumor-bearing mice were treated with a combination of irradiation and anti-PD1 antibody. Results showed combination treatment significantly delayed the tumor growth and decreased tumor burden over either treatment alone (Fig. 5A and B). Further analysis of tumor tissues showed that the combination treatment increased CD8^+^ TILs, IFNγ and granzyme B in tumor tissues (Fig. 5C and D).

## DISCUSSION

Abnormalities in multiple signal pathways in cancer and the resistance to chemotherapeutics may be pivotal issues that spur the great need to develop new treatments [17]. Immune checkpoint blockade has emerged as an important and effective form of immunotherapy against human cancer [18]. However, despite the clinical potential of these inhibitors, most patients either do not respond in the clinical trials or develop resistance. To overcome these limitations, methods to improve the efficacy would be advantageous [19]. It has been shown that combinations of radiation therapy and immunotherapy can produce more effective anti-tumor responses than single treatments. The connections between radiation and immune therapies have been intensively studied in cancer research programs.

Radiation therapy is used to treat cancer due to its direct toxic effects on tumor cells, and its ability to affect tumor cell immune response is increasingly recognized, through inducing the death of cancer cells and releasing new antigens to various components of the immune system, thereby improving the initiation and activation of effector T cells [20]. Radiation-induced cell death released proteins as immunological danger signals to present antigens to cytotoxic T cells via toll-like receptors (TLR) on DCs, resulting in the release of cytokines and the attraction of T cells into the irradiated tumor [21]. Furthermore, radiation therapy increased expression of surface molecules of irradiated cancer cells and rendered them more susceptible to cytotoxic T cell-mediated killing [22]. In addition, type I IFN can activate DCs [23] and CD8^+^ T cells, and promote the development of CD4^+^Th1 [24], which is necessary for radiation therapy to cause an anti-tumor immune response [25]. In this study, we investigated the gastric tumor response to radiation therapy, based on the Sting signal, *IFNB1, TNFα, CXCL-9* as well as *IL-10* gene expression, infiltration of DCs, Th and Teff cells in the tumor microenvironment, and PD-1/PD-L1 expression after radiation therapy.

Vascular endothelial cells in the tumor microenvironment are damaged and dysfunctional after irradiation treatment, and tissue hypoxia and other effects may cause resistance to radiation therapy [26, 27]. The Sting pathway has been identified as an important mechanism by which the innate immune system is capable of recognizing tumors, in order to initiate a type I interferon (IFN-I)-driven inflammatory program that stimulates DC cross-presentation of tumor antigens, ultimately leading to mobilization of tumor-specific CD8^+^ T cells [28–30]. In MFC cells, Sting protein and *IFNB1, TNFα* as well as *CXCL-9* genes significantly increased after irradiation treatment, implying that the Sting signaling pathway was activated by DNA damage caused by radiation therapy [31]. Early studies showed the Sting signaling pathway was mainly carried out in DCs, macrophages and other antigen-presenting and hematopoietic cells [32]. However, through exogenous double stranded DNA (dsDNA) stimulation, the Sting signaling pathway could be expressed in many cancer cells. Our results showed that the expression of Sting protein was upregulated after radiation in MFC tumor tissues. The irradiation treatment also increased the marker of Sting activation and IFN-I responses (CXCL-9), T cell infiltration and function (CD4, CD8, IFNγ, granzyme B), and decreased immunosuppression expression (IL-10). This finding may be useful for further studies on how to inhibit tumor growth through activating the Sting signaling pathway.

Radiation therapy increased immune cell infiltration in tumor tissues, and various cellular molecules were up-regulated [33, 34]. Our results confirmed that irradiation treatment activated immune responses and increased lymphocytic infiltration in tumor tissues in an MFC mouse model. However, the outcome of irradiation treatment was not influenced by immune infiltration, shown by the lack of tumor growth control, which may be due to the up-regulated expression of PD1/PD-L1 after irradiation treatment. The increased immune cells inside tumor tissues could not exert anti-tumor effects because of the PD1/PD-L1 pathway, suggesting a potential therapeutic approach involving radiation therapy combined with immunotherapy, such as anti-PD1 antibody, to release the immune system to eliminate cancer cells. Thus, radiation therapy combined with immunotherapy may yield better outcomes than either alone, while reducing the dose of radiation therapy and alleviating the toxic side-effects.

Patients with cancer have got benefit from the successes of radiation therapy and immune checkpoint-blockade immunotherapy to raise median survival of melanoma patients with brain metastasis from 4.9 to 21 months [35–38]. However, whether the radiation and immunotherapy have synergistic effects was not addressed. In this study, either irradiation or anti-PD1 blockade alone was not able to significantly delay tumor growth, however, the combination treatment induced systemic immune responses and decreased the tumor burden, indicating these two treatment strategies worked together.

This study investigated effects of radiation on gastric tumors and demonstrated an enhanced immune response in a radiation therapy-insensitive gastric tumor model. Our findings highlighted the benefit of combining radiation therapy with immune checkpoint inhibitors against radiation therapy-insensitive gastric cancer, suggesting synergistic effects of these two treatment modalities and providing the basis for their use in clinic.

## CONFLICT OF INTEREST

None declared.

## Supplementary Material

supplementary_figure_rraa077
