# Supplementary figures and images for: Radiation therapy enhanced therapeutic efficacy of anti-PD1 against gastric cancer
Source: J Radiat Res. 2020 Sep 22;61(6):851–9. doi: 10.1093/jrr/rraa077 (PMC7674687; doi:10.1093/jrr/rraa077)

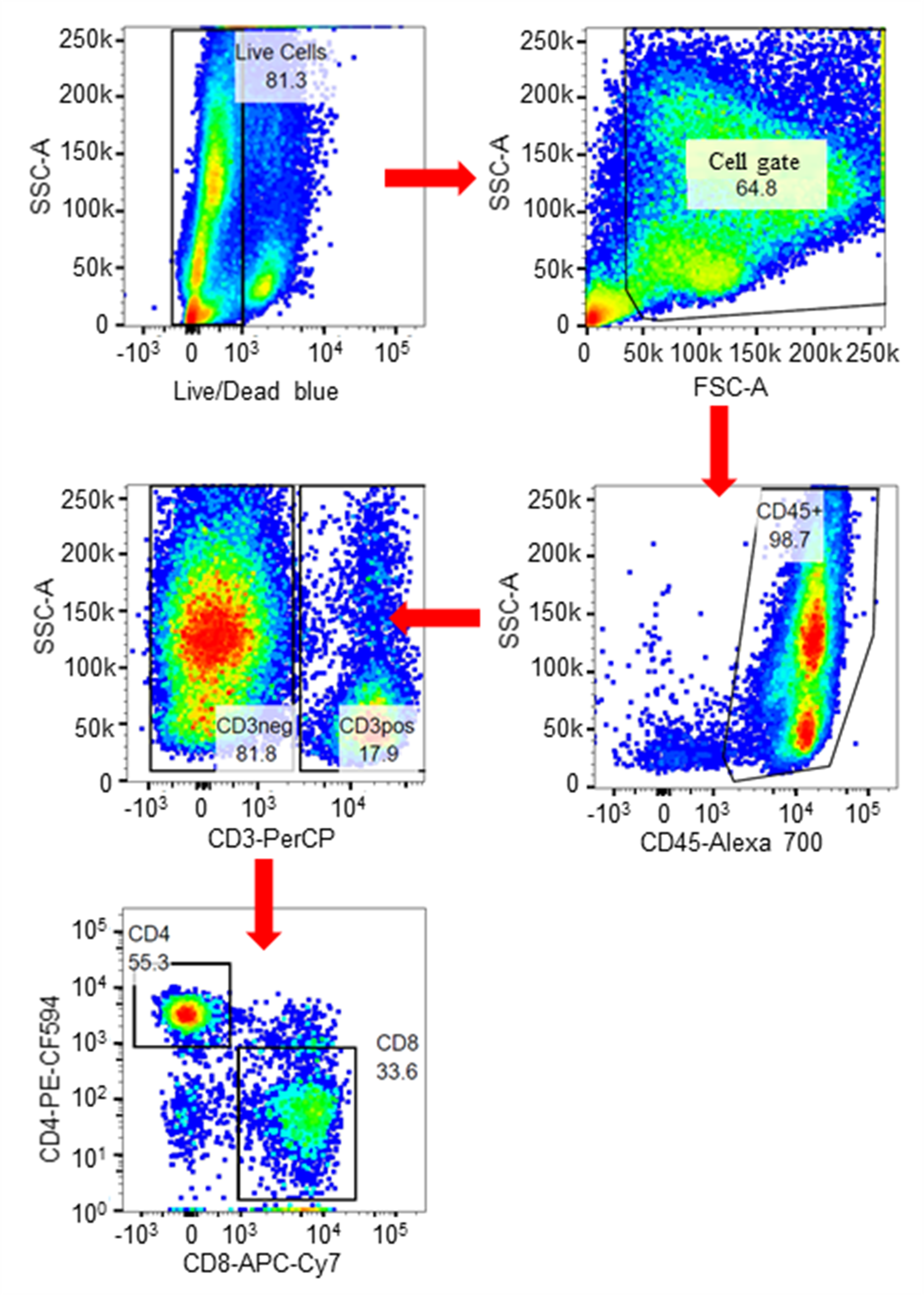

Supplement: supplementary_figure_rraa077 [file supplementary_figure_rraa077.png]
